# Assessment of ultrasonic data of signals backscattered by mortar using the principal component analysis

**DOI:** 10.1016/j.dib.2021.106741

**Published:** 2021-01-14

**Authors:** Hicham Lotfi, Driss Izbaim, Hassan Bita, H. Mesbah, H. Banouni

**Affiliations:** a Higher School of Technology-Laâyoune - (ESTL) Morocco; b Laboratory of Metrology and Information Processing, Ibn Zohr University, Agadir, Morocco

**Keywords:** Non Destructive Testing, Ultrasonic velocity, Mortar, Principal component analysis (PCA)

## Abstract

In this paper, we present the assessment of ultrasonic parameters of signals backscattered by mortar using the Principal Component Method. The measurement has been performed by the ultrasonic reflection technique using a transducer with a central frequency 0.5 MHz. The samples examined consist of specimen mortar mixtures prepared with ratio cement/sand (c/s = 0.5) and water/cement (w/c = 0.65) and studied at three temperatures 25 °C, 32 °C and 42 °C. The projection of the data showed that more than 93% of the information is retained and presented in 2D space. The correlation between Acoustic Impedance and Ultrasonic Velocity at 25 °C, 32 °C and 42 °C are 96.95%, 99.98%, and 99.98% respectively. The data are associated with the research article “Ultrasonic Characterization and Hardening of Mortar Using the Reflection Technique” (H.LOTFI and al.).

## Specifications Table

**Table:** 

Subject	Materials Science
Specific subject area	Materials Science, Materials Processing, Material Characterization
Type of data	Figures
How data were acquired	The experimental protocol compound a digital oscilloscope, a pulse generator linked to a transducer with a central frequency 0.5 MHz. Microcomputer for acquisition of data. Software: Labview for acquisitions of data. Excel and Minitab for analysis and treatment of data.
Data format	Raw, analyzed.
Parameters for data collection	All the experiments of characterization of the durability of mortar were carried out under the same conditions (same sand, same size, and w/c and c/s mass ratio). The setting of the temperature experiment is done by using thermostatically controlled tank.
Description of data collection	Primary data (i.e. raw materials, operating data) were collected via Labview software and via an acquisition card. Data was processed using Excel and Minitab 19, for purposes of data analysis and diagram presentation. To access to *.RES* files, they are opened with Bloc-notes.
Data source location	Faculty of Sciences Agadir, Region: Souss Massa Daraa, Morocco (30 °24′25.9"N 9 °32′42.7"W)
Data accessibility	Data identification number: Direct URL to data https://data.mendeley.com/datasets/zgnrtm6pvw/2
Related research article	H. Lotfi, B. Faiz, A. Moudden, A. Menou, I. Izbaim, G. Maze and D. Decultot: Ultrasonic Characterization and Hardening of Mortar Using the Reflection Technique. High Temperature Materials and Processes https://doi.org/10.1515/HTMP.2009.28.4.263

## Value of the Data

• These data can be used in this study of the improvement of the resistance and the quality of mortars, also to evaluate the effectiveness of the ultrasonic technique on cementitious materials.
• This data helps others to understand the effect of temperature and sand microstructure of sand on the evolution of ultrasonic parameters during the setting of mortars.
• The data can be used to compare the effect of other temperatures and mass ratios (water/cement and cement/sand), in order to solve problems of faulty setting mortars that cause cracks in building walls and structures.

## 1 Data Description

**Fig. 1:**
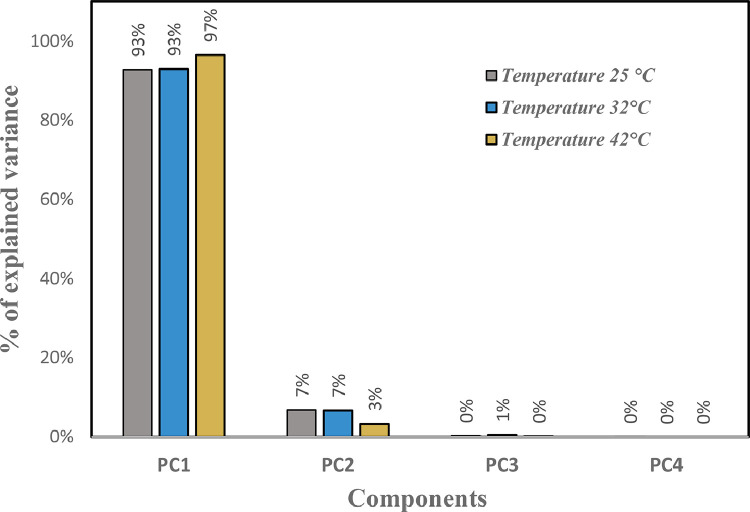
Distribution of the information retained on the first four variance axes.

**Fig. 2:**
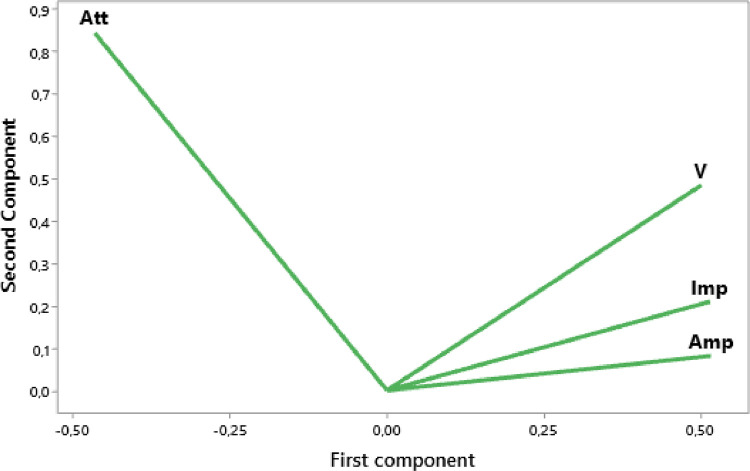
Variables projection in (PC1 PC2) plane for the temperature of 25 °C.

**Fig. 3:**
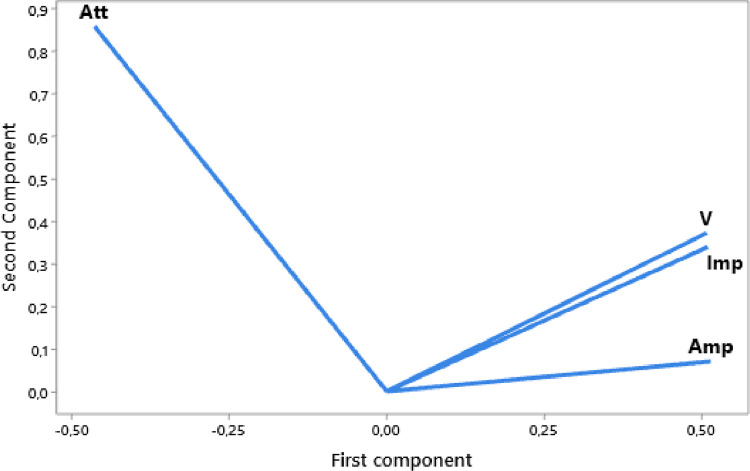
Variables projection in (PC1 PC2) plane for the temperature of 32 °C.

**Fig. 4:**
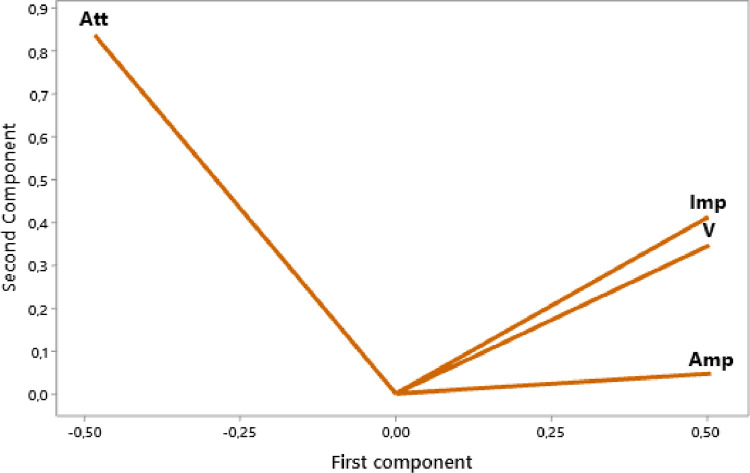
Variables projection in (PC1 PC2) plane for the temperature of 42 °C.

**Fig. 5:**
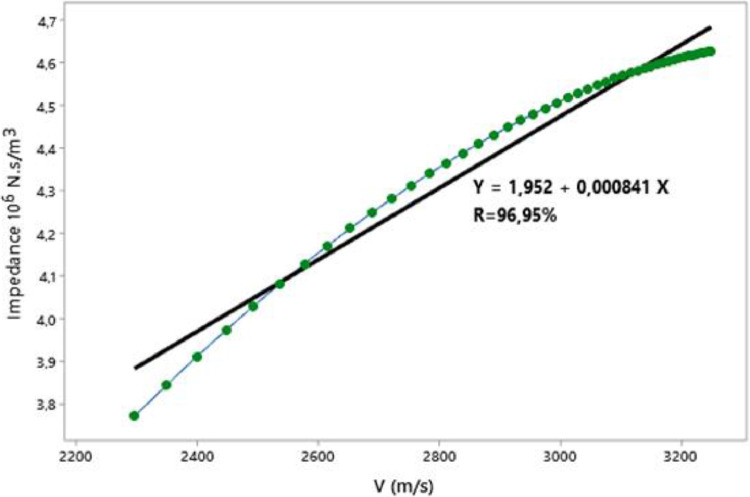
Linear regression for Velocity and acoustic impedance for T=25 °C.

**Fig. 6:**
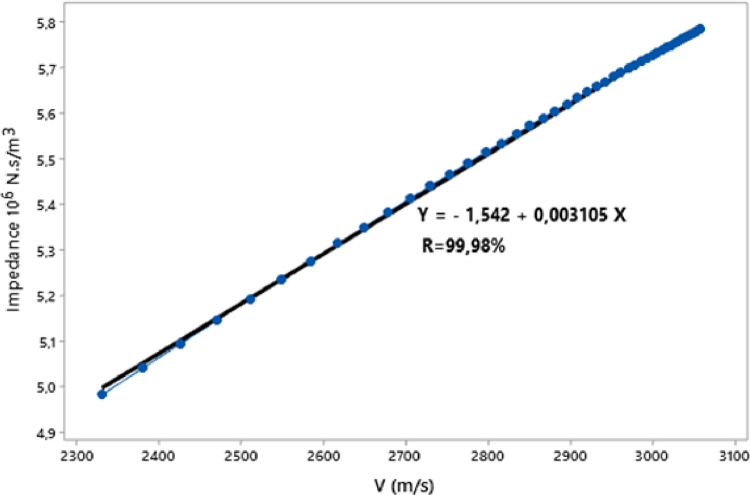
Linear regression for Velocity and acoustic impedance for T=32 °C.

**Fig. 7:**
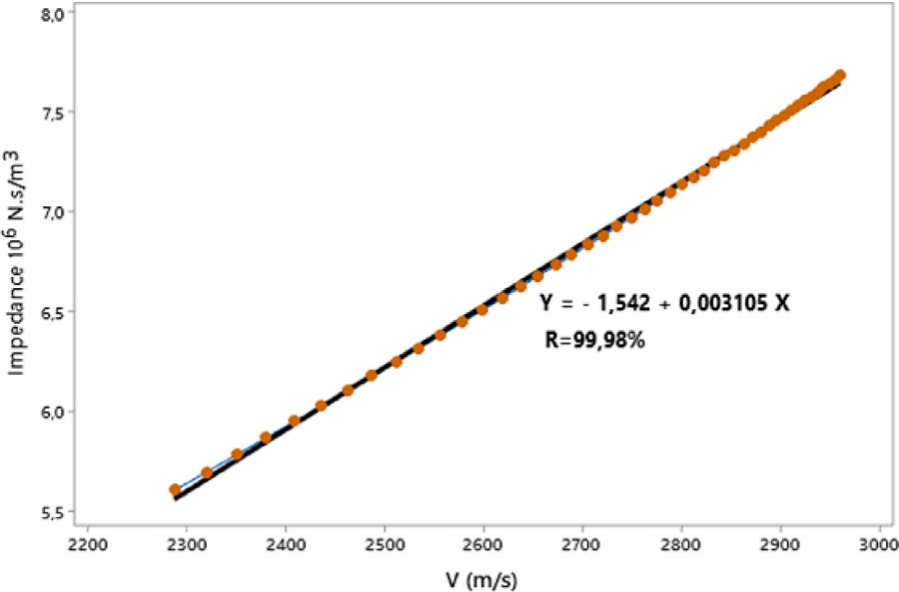
Linear regression for Velocity and acoustic impedance for T=42 °C.

The raw experimental data presented in this article represents the temporal signals that were obtained by ultrasonic acquisition every 60 minutes, for the structures of the mortars prepared with the grains of sand 0.315 mm and for the mass ratios (c / s = 0.5 and w / c = 0.65) and for temperatures 25, 32, and 42 °C. Also, the data obtained by application of Principal Component Analysis (PCA) method are provided to study the correlation between the viscoelastic parameters measured during the propagation of ultrasonic waves in the structures of mortars. Fig. 1 represents projection in 2D space of the data obtained, for the first two dimensions PC1 and PC2, using principal components analysis. In Fig. 2, we represent the projection of variables attenuation, velocity, acoustic impedance, and amplitude in the plane generated by the components (PC1 PC2) for the temperature of 25 °C. Fig. 3 illustrates the projection of the same variables in-plane (PC1 PC2) for the temperature of 32 °C. For the temperature of 42 °C, the projections of variables are presented in Fig. 4. In Fig. 5, we represent linear regression between the Velocity and Acoustic Impedance parameter of signals backscatter by mortar prepared at the temperature of 25 °C. Fig. 6 shows the correlation between Acoustic Impedance and Ultrasonic Velocity for the specimen prepared at 32 °C. For mortar realized at 42 °C the linear regression between Velocity and Acoustic Impedance is presented in Fig. 7. For the raw data they are grouped in a folder named “*Acquisition of Data Mortar size 0.315 mm”* contains all the data of the temporal signals acquired for the temperatures 25, 32 and 42 °C. In addition, a file named *“Viscoelastic Parameters and Data ACP Analysis*” contains the data present in all figures by applying the Principal Component Analysis (PCA) method. Raw data available in Mendeley Data http://dx.doi.org/10.17632/zgnrtm6pvw.2

## 2 Experimental Design, Materials and Methods

The experimental protocol used to follow the evolution of mortars in real time is based on the reflection method, using the transducer with a central frequency 0.5 MHz as a transmitter and a receiver of ultrasonic waves. Mortar to be characterized is contained in a parallelepiped vessel immersed in a thermostaed water. The pulse generator sends an electrical pulse to the transducer, which is transformed into an acoustic wave. After propagation in water, the incident acoustic wave is partly reflected on the vessel and partly transmitted in the mortar sample through the vessel. The reflected acoustic signal composed of a series of echoes is converted into an analogy electrical signal, and is amplified and digitalized by a digital oscilloscope. The data are sent through an interface cable to a personal computer that calculated the different viscoelastic parameters [1]. The first parameter, ultrasonic velocity, is defined as the ratio of the fly time between the A2 and A3 echoes to the thickness of the mortar L. A_2_ is the echo which corresponds to the reflection on the interface between the second face of the Plexiglas plate and the mortar, and A_3_ is the echo corresponding to the reflection on the interface between the mortar and the first face of the glass plate [1]. The second parameter is the acoustic impedance Z, which characterizes the quality of the material to transmit the ultrasonic waves. It is calculated by the product of the mortar density and the ultrasonic velocity of the wave in the mortar. The third parameter is the peak-to-peak amplitude of the A_2_ echo. This parameter is measured after its isolation in the time domain by using a filtering method. The last viscoelastic parameter is the attenuation coefficient. It is related to the ratio of the spectral amplitudes A_2_( ν) and A_3_(ν) and the thickness of the mortar L; the A_2_( ν) and A_3_(ν) are calculated by using the Fourier transform of the temporal echoes A_2_ and A_3_.

## CRediT Author Statement

**Driss IZBAIM**: Conceptualization, Methodology, Software; **Hicham LOTFI**: Data curation, Writing- Original draft preparation; **Hassan Bita**: Investigation, Reviewing and Editing.

## Declaration of Competing Interest

The authors declare that they have no known competing financial interests or personal relationships, which have, or could be perceived to have, influenced the work reported in this article.

## Appendix Supplementary materials

Image, application 1
