# Supplementary material for: Assessment of ultrasonic data of signals backscattered by mortar using the principal component analysis
Source: Data Brief. 2021 Jan 14;34:106741. doi: 10.1016/j.dib.2021.106741 (PMC7820378; doi:10.1016/j.dib.2021.106741)
Supplement: Supplementary file 1 [file mmc1.pdf]

Ultrasonic Characterization and Hardening of Mortar Using the Reflection Technique

H. Lotfi[1*], B. Faiz[1], A. Moudden[1,2], A. Menou[3],
I. Izbaim[1], G. Maze[4] and D. Decultot[4]

[1] *Laboratoire Métrologie et Traitement de l'Information Faculté des Sciences Agadir Morocco*
[2] *Ecole Supérieur de Technologie Agadir, Morocco*
[3] *ONDA/AIMAC, Aéroport Med V, Casablanca Morocco*
[4] *Laboratoire Ondes et Milieux Complexe Université Le Havre France*

(Received July 20, 2009: final form September 13, 2009)

ABSTRACT

In this study we described a Non-Destructive Testing (NDT) method which can be used to characterize some samples of mortars and the effect of the microstructure of sand in their hardening. Samples were manufactured using the same water/cement ration(w/c) 0.65, and cement/sand ratio (c/s) 0.5 in order to simulate the attenuation, acoustic impedance and ultrasonic velocity. The characterization was performed by an ultrasonic reflection technique using a transducer with central frequency 0.5 MHz. It is shown that the sand particle size exercises significant influence on the evolution of attenuation, ultrasonic velocity and acoustic impedance. The first two parameters give information about the average state of hardening of the mortar itself. The third parameter, acoustic impedance, indicates the state of hardening of the mortar and the interface with vessel.

Keywords: Non-Destructive Testing; Reflection technique; Attenuation; Ultrasonic velocity; Mortar.

1. INTRODUCTION

The hardening of cementing materials is accompanied by several mechanical phenomena, thermal and physicochemical. The beginning of last synthesis of work century shows the first devices of the measurement of hardening, which are mainly based on Vicat apparatus. The study of the hardening was subsequently approached using other techniques based on the measurement heat release during hydration, electronic microscopy and electric conductimetery. Currently the Vicat test represents the standard test of the follow-up of the hardening of cementitious materials. However the result obtained from this test is insufficient to judge, for example, the time during in which, the material is fresh or hardened. This short outline shows that the methods of characterization by ultrasound has not advanced in civil engineering since the work of Vicat in terms of standardization. In this context, we present a non-destructive investigation based on the emission of the ultrasonic waves following the evolution of viscoelastic parameters.

The measurement of the ultrasonic wave velocity was used to characterize hardening and setting of cementitious materials /1,4/. Characterizing the hardening of mortar by ultrasonic techniques is very attractive because these techniques are not destructive and permit the observation of the hardening in real time had used a reflection method to evaluate material properties of isotropic materials /5-6,20/.

In this paper a reflection pulse echo method is studied in order to analyze the speed of propagation of

Brought to you by | Purdue University Libraries
Authenticated
Download Date | 6/3/15 8:47 PM

properties of echoes extracted from the reflected echo wave form

2. EXPERIMENTAL PROCEDURE

2.1 Ultrasonic method and equipment

The experimental reflection pulse echo method is described in Figure 1. The main parts of the experimental set up are shown. Mortar to be characterized is contained in a parallelepiped vessel ($50\times100\times35\times10^{-9}m^3$) immersed with a transducer in thermostaed water. The broadband transducer is used successively as emitter and receiver, and is excited by an impulse generator (5075 PR Model, Panametrics, Sofranel, France). The transducer is a panametric's (V302) transducer with a central frequency 0.5 MHz. The pulse generator sends an electrical pulse to the transducer, which is transformed into an acoustic wave. After propagation in water; the incident acoustic wave is partly reflected on the vessel and partly transmitted in the mortar sample through the vessel.

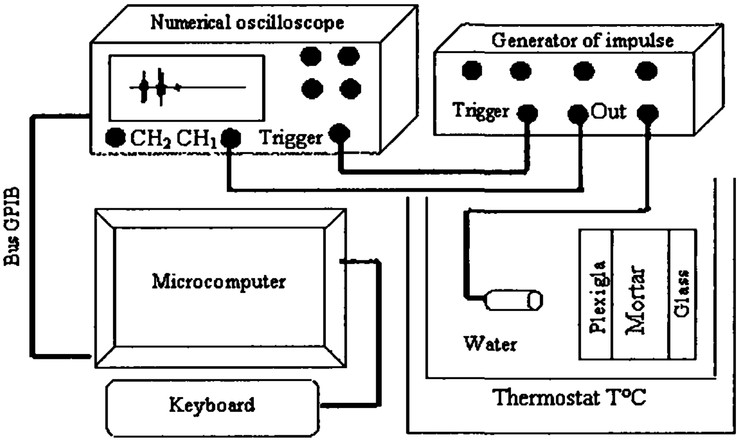

Fig. 1: Experimental setup used the reflection method

The reflected acoustic signal composed of a series of echoes is converted into an analogy electrical signal, and is amplified and digitalized by the Lecroy digital oscilloscope (300 MHz bandwidth, 9310M model). The data are sent through an IEEE 488 interface to a personal computer that calculated the different viscoelastic parameters of the mortar in order to avoid superposition of different echoes, the thickness D_p, L and D_g respectively of plate 1 (Plexiglas), mortar and plate 2 (Glass) are chosen as indicated in Table 1. The experimental method consists of the analysis of different reflected echoes.

Table 1

Thickness of plate 1, mortar and plate 2

	D_p (Plexiglas)	L (Mortar)	D_g (Glass)
Thickness	0.02 m	0.015 m	0.003 m

The geometry of the structure is shown in Figure 2. The incident acoustic wave is considered as a plan wave. Part of the ultrasonic energy is transmitted into the mortar through plate 1 and, after viscoelastic propagation in the mortar; part of the energy is reflected from plate 2 and returns to the same transducer used as receiver.

Brought to you by | Purdue University Libraries
Authenticated
Download Date | 6/3/15 8:47 PM

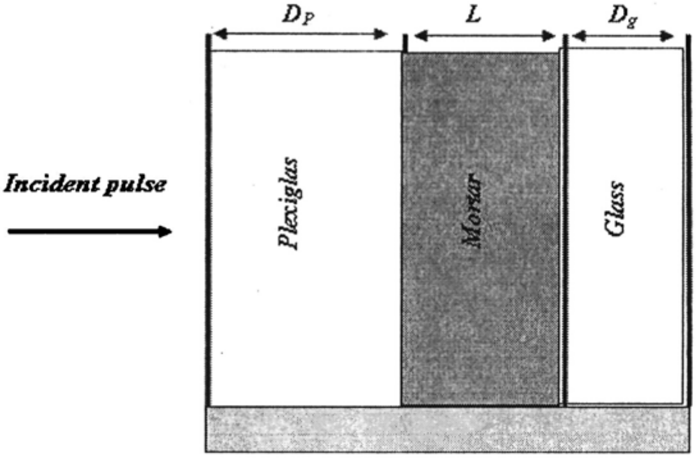

Fig. 2: Problem geometry of the mortar

Figure 3 shows the different paths taken by the ultrasonic wave. Each path provides an echo which is labelled E_n.

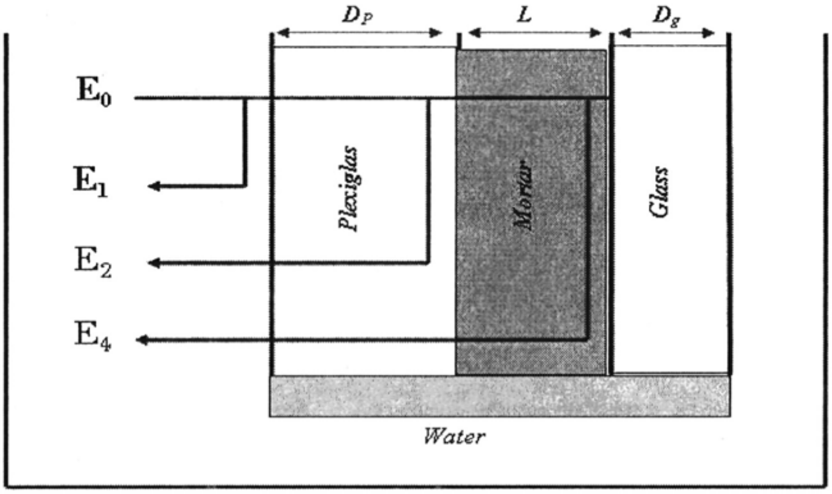

Fig. 3: Different paths of propagation in mortar

Figure 4 presents the signal composed of echoes E_1, E_2 and E_4 reflected by the vessel enclosing the mortar using the 0.5 MHz central frequency transducer. The first E_1 echo is reflected on the bottom surface of plate 1 (Plexiglas). This is not important because it is not used in this study. The second echo E_2 corresponds to the reflection on the interface between the second Plexiglas face and the mortar sample enclosed in the vessel. The last echo E_4 corresponds to the reflection on the interface between the mortar and the first face of plate in glass (plate 2).

The choice of the vessel material (Plexiglas) is done in relation with the characteristics of the mortar and its thickness (D_p=0.02 m) is chosen in order to sufficiently attenuate echoes (reflection in plate 1). In this study, only spectral properties of echoes E_2 and E_4 was analyzed to determine viscoelastic parameters of the mortar in real time.

Brought to you by | Purdue University Libraries
Authenticated
Download Date | 6/3/15 8:47 PM

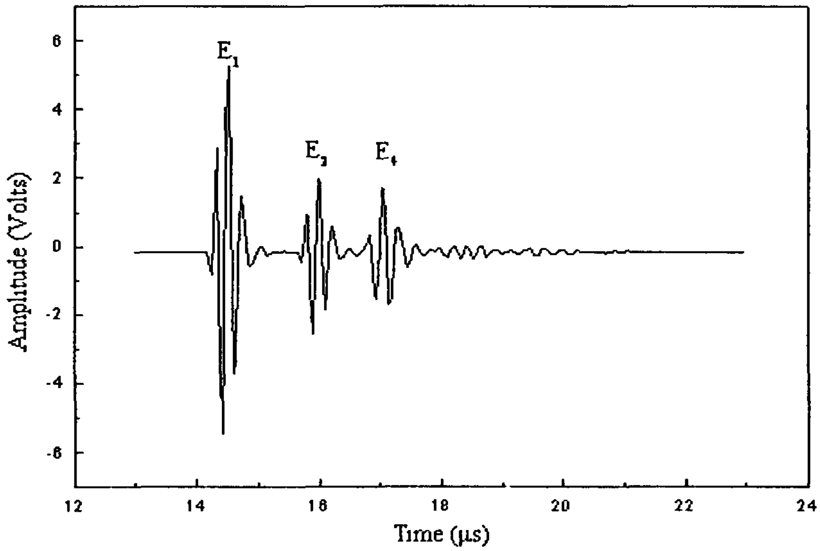

Fig. 4: Reflected echoes by the mortar with 0.5MHz transducer, time domain

2.2 Samples

Portland cement mortar with water/cement (w/c) ration (0.65) and cement/sand (c/s) ratio (0.5), sand aggregates with diameter (2, 2.5, 3.15 and 5 $10^{-4}m$) were investigated. Portland cement CPJ 45 produced in Agadir, Morocco, was used as the cementitious materials and their chemical compositions and physical properties are summarized in Table 2. In the sand used was a river sand of OUED SOUSS with mass volume absolute ($2.63\times10^6 Kg/m^3$), mass volume apparent ($1.46\times10^6 Kg/m^3$). The mortar were cured at isothermal conditions at 315K through the duration of the experiments , the temperature chosen according to the climate of the regions in south of Morocco.

Table 2

Chemical compositions and physical properties of cement CPJ 45

	Chemical composition (mass %)											Physical properties
	insoluble	SiO_2	Al_2O_3	FeO_3	CaO	MgO	SO_3	K_2O	P_2O_5	Cl^-	LI*	Blaine m^2/Kg
cement	*11.8*	*16.7*	*5.48*	*2.8*	*51.8*	*3.1*	*2.7*	*0.8*	*0.28*	*0.02*	*4.7*	*409.2*

*(LI) : loss on the ignition

3. RESULTS AND DISCUSSION

3.1 Attenuation measurement

The determination of viscoelastics parameters has been the subject of several studies /7,8/, which enables us to obtain the elastic parameters of the mortar enclosed within a parallelepiped vessel, this determination is achieved by the analyse of the time signal (Figure 4) and frequency signal (Figure 5). The attenuation coefficient is determined by measuring the reduction in amplitude of an ultrasonic wave, which has travelled distance L of mortar , and can be written for the frequency υ as /7,9/:

Brought to you by | Purdue University Libraries
Authenticated
Download Date | 6/3/15 8:47 PM

$$\alpha(\upsilon) = -\frac{1}{2L}\ln(\frac{E_4}{E_2}\chi_{ref})$$

With: $$\chi_{ref} = \left|\frac{(Z_{mor} - Z_{pg})(Z_{pg} + Z_{mor})(Z_{mor} + Z_{glass})}{4Z_{mor}Z_{pg}(Z_{glass} - Z_{mor})}\right|$$

The echoes E_2 and E_4 are isolated separately through a time-based filtering program established in the personal computer. Their corresponding amplitude spectral are denoted $E_2(\upsilon)$ and $E_4(\upsilon)$, Z_{mor} are the acoustic impedance in the mortar, Z_{pg} is the impedance of the Plexiglas plate calculated from the relation $Z_{pg}=C_{pg}.\rho_{pg}$, C_{pg} is the velocity of Plexiglas (2680 m/s) and ρ_{pg} its density ($1.2\times10^9 Kg/m^3$), and Z_{glass} are the acoustic impedance of the glass plate its value is $1.331\times10^7 N.s/m^3$. The difference of amplitudes of the transmitted and received pulse signals gives an indication of the signal attenuation through the thickness of mortar and has been used to evaluate the quality and hardening of mortar.

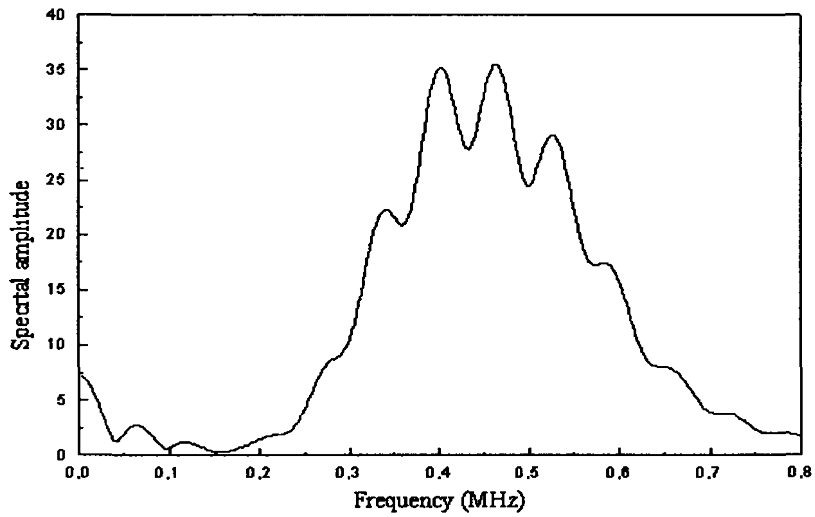

Fig. 5: Spectral amplitude of the E_2 echoes and E_4 , frequency domain

Acoustic impedance and attenuation were determined as function of time for respective sand particle size 2, 2.5, 3.15 and 5 $\times10^{-4}m$ using a transducer with frequency central 0.5 MHz. The attenuation with frequency for such materials containing aggregates has been reported in /1,10-13/.Comparison between different sand particle size sharing the same parameter w/c and s/c revaluated that mortar realized with $3.15\times10^{-4}m$ is more attenuative, the behaviour is more clear about 20h, as can be seen in Figure6. The difference in attenuation can be attributed to the different degree of particle seize of sand. An important outcome of the result above is the different level of attenuation between mortar realized by sand particle size $3.15\times10^{-4}m$ and mortar realized by sand particle size $5\times10^{-4}m$ related to their inhomogeneity.

Brought to you by | Purdue University Libraries
Authenticated
Download Date | 6/3/15 8:47 PM

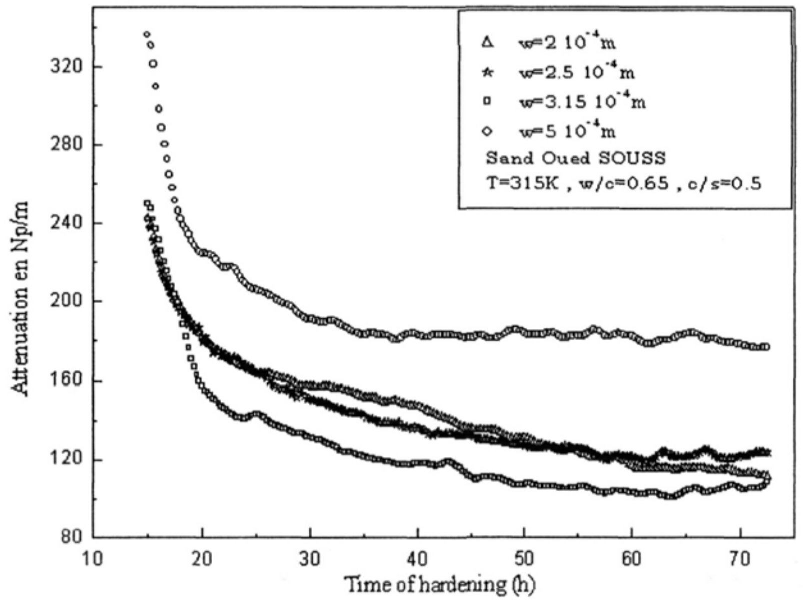

Fig. 6: Evolution of attenuation of ultrasonic wave for various sand sizes

3.2 Ultrasonic velocity measurement

One of the most important viscoelastic parameters for following the hardening, in real time, of mortar and other materials is the ultrasonic velocity. Velocity v applies in general at the known interrelationship of $v = 2L/\Delta t$ with travel time Δt which is the variation of time between echoes E_1 and E_2, and travel path L, number 2 refer to double path of signal backscarted by the mortar inclosing in the vessel (method of echoes). Starting from the analysis of the profiles of ultrasonic velocity, three important stages can be distinguished as shown in Figure 7.

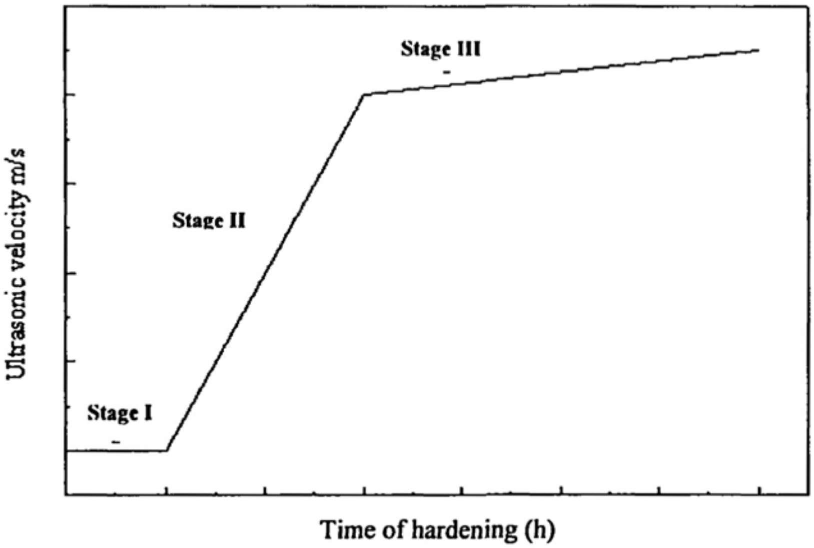

Fig. 7: Stages characterize the evolution of ultrasonic velocity

Brought to you by | Purdue University Libraries
Authenticated
Download Date | 6/3/15 8:47 PM

Stage I: This stage proceeds in the interval of the time of 0 at 15h, in this period the ultrasonic velocity is relatively small what is in agreement with other authors /14-16, 22/. This phenomenon is caused by the air generated in the paste of the mortar, and which is generally due to two reasons, the first is that a certain quantity of the bubbles of air in the mixture, the second which there is a number of bubble of air are formed in the paste during malaxation /17/. Therefore the existing air inside the paste plays an important part in the evolution of ultrasonic velocity.

Stage II: In this stage the ultrasonic velocity starts to increase, this is caused by air between the sand grains and the mixture changes position during the process of hydration, this air generated migrates towards the surface of the mortar from where the generated quantity is reduced. In addition the phases of solid become increasingly connected and the mortar transforms from suspension state of into elastic and porous. During this stage it is clear that the ultrasonic velocity is controlled by the reduction of the bubbles of air /18/.

Stage III: A light increase of ultrasonic velocity is observed, from where the influence of the bubbles of air is very weak, and water in the structure is completely consumed at the period of hardening of mortar. It's clearly that the wave velocity increase with age, it is assumed in /19, 21/ that the pulse velocity increases with the increase of mortar strength.

We notice that the curves shown in Figure 8, have the same evolution, and the velocity increases strongly in the interval of time (15h to 50h), after 50h the velocity becomes almost constant, it is the period of hardening of the mortar. Thus for the same temperature and the same mass ratios of cement/sand and water/sand the velocity of the signals backscarted is very important for the mortar containing sand particle size $3.15\times10^{-4}m$

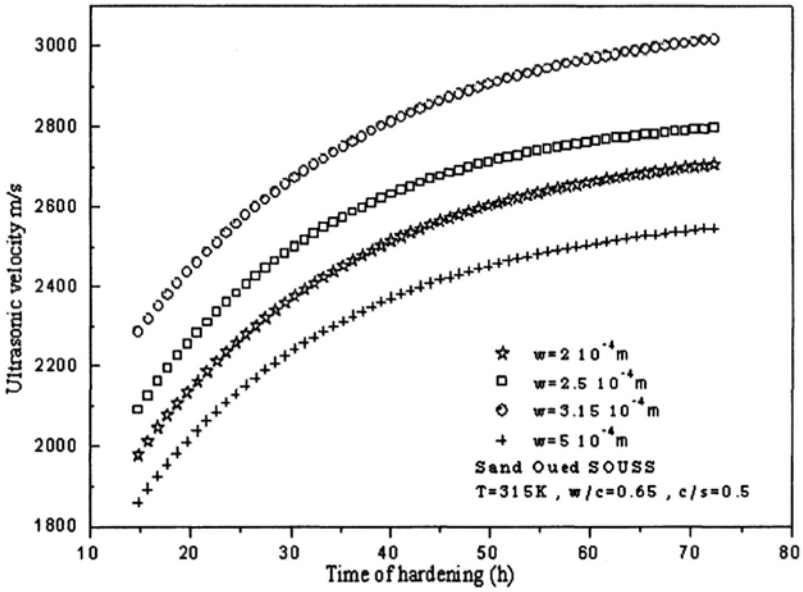

Fig. 8: Evolution of ultrasonic velocity for various sand sizes

Brought to you by | Purdue University Libraries
Authenticated
Download Date | 6/3/15 8:47 PM

4. CONCLUSION

In the present paper results from an experimental study of ultrasonic reflection technique (Non-destructive testing) measurements on mortar's samples enclosed in a vessel of Plexiglas and glass, are described. This technique is based on the analysis of ultrasonic signal backscarted by the mortar where the hydration, is using a transducer of central frequency 0.5MHz.

The following conclusions can be given.

1. Experimental investigations have shown that sand particle size influences velocity measurements.
2. Using the comparison between different pastes of mortars, the sand particle size seems to have a more definite impact on the attenuative behaviour than on the ultrasonic velocity.
3. We also noted that the presence of the impurities in the small particles of sand, and their mineralogical character inhibits the hardening of the mortars. In this same way for the large size, ultrasonic velocity are low due to the interconnection between grains, thus formation of the anomalies and cracks in the structure of mortar.
4. There exist an exponential relationship between the acoustic impedance Z (strength of mortar) and ultrasonic velocity V of ultrasound propagation.

ACKNOWLEDGEMENTS

The research presented in this paper was funded by the Laboratory of Metrology and Data Processing, Agadir, Morocco, and School University of Technology (EST) Agadir, Morocco their support is gratefully acknowledged.

REFERENCES

1. T.P. Philippidis and D.G. Aggelis, *Ultrasonics*, **43** ,584-595 (2005).
2. D.G. Aggelis, D. Polyzos and T.P. Philippidis, *Journal of the Mechanics and Physics of solids,* **53** , 8570883 (2005).
3. Thomas Voigt, Guang Ye, Zhihui Sun, Surendra P. Shah and Klaas van Breugel, *Cement and Concrete Research,* *35*, 858-866 (2005).
4. H.K. Lee, K.M. Lee , Y.H. Kim, H.Yim and D.B.Bae, *Cement and Concrete Research*, **34**, 631-640 (2004).
5. A.R. Selfridge, *IEEE Trans.sonics Ultrason.*, SU-32, 381-394, (1985)
6. B.Faiz, G.Maze, D.Deculto, E.Aassif and M.Ezzaidi, *IEEE Trans. Ultrasonics, Ferroelectrics ,and frequency control,* **46**, (1), 188-196 (1999).
7. R.A.Kline, *J. Acoust. Soc.Am.*, **76**, 498–504 (1984).
8. D.K. Mak, *Br. J. Non-Destruct. Test.* **35** 443–449 (1993).
9. D.K.Mak, *J. Non-Destruct. Test.* **33** 441–449 (1991).
10. Y.H. Kim, S. Lee and H.C. Kim, *J. Phys. D: Appl. Phys.*, **24**, 1722–1728 (1991).
11. E.N. Landis and S.P. Shah, *J. Eng. Mech.—ASCE* **121** (6) 737–743(1995).
12. P.A. Gaydecki, F.M. Burdekin, W. Damaj, *Meas. Sci. Technol.*, **3**, 126–134 (1992).
13. L.J. Jacobs and J.O. Owino, *J. Eng. Mech.—ASCE* **126** (11) , 1124-1130 (2000).
14. D.G. Aggelis, S.V. Tsinopoulos, J.T. Verbis, T.P. Philippidis and D. Polyzos, On the wave propagation in concrete, in *Scattering and Biomedical Engineering Modeling and Applications,* edited by D. Fotiadis and C. Massalas,World Scientific, Singapore, (2002), p. 175– 184.
15. J. Keating and D.J. Hannant, *Cement and Concrete Research,*. **19**, 715–726 (1989).
16. C.M. Sayer and A. Dahlin, *Adv. Cem. Based.Mater.*, **1**, 12– 21(1993).
17. G. Ye, K. van Breugel and A.L.A. Fraaij, *Cement and Concrete Research*, **33**, 233–239(2003).
18. S.J. Lokhorst, *Deformational behavior of concrete influenced by hydration-related changes of the microstructures, Research report* 25.5.99-5, Delft University of Technology, (1999).
19. J. Blitz and G.Simpson, *Ultrsonic methods of non – destructive testing*, Chapman & Hall, London, (1996).
20. M. Ezzaidi, A.Mouddcn , D.Deculto and G.Maze, *NDT& E international* **35**, 433 436(2002).
21. G. Ye, P. Lura, K. van Breugel, A. L. A. Fraaij , *Cement & Concrete Composites* **26**, 491–497(2004).
22. M. Ghrici, S. Kenai, M. Said-Mansour, *Cement & Concrete Composites* **29**, 542–549(2007).

Brought to you by | Purdue University Libraries
Authenticated
Download Date | 6/3/15 8:47 PM
